# Acute kidney injury in children and adolescents with diabetic ketoacidosis managed with aggressive initial volume resuscitation

**DOI:** 10.1016/j.clinsp.2026.101029

**Published:** 2026-07-08

**Authors:** Danielle Saad Nemer Bou Ghosn, Thais Della-Mana, Isabel de Siqueira Ferraz, Claudio Schvartsman, Sylvia Costa Lima Farhat

**Affiliations:** a Emergency Department, Instituto da Criança e Adolescente do Hospital das Clínicas da Faculdade de Medicina da Universidade de São Paulo, São Paulo, SP, Brazil; b Endocrinology Division, Instituto da Criança e Adolescente do Hospital das Clínicas da Faculdade de Medicina da Universidade de São Paulo, São Paulo, SP, Brazil; c Paediatric Intensive Care Unit, Hospital de Clínicas da Universidade de Campinas, Campinas, SP, Brazil; d Hospital Israelita Albert Einstein, São Paulo, SP, Brazil; e Laboratory of Experimental Air Pollution, LIM05, Faculdade de Medicina FMUSP, Universidade de São Paulo, São Paulo, SP, Brazil

**Keywords:** Diabetic ketoacidosis, Child and adolescent, Fluid therapy, Acute kidney injury, Chlorides

## Abstract

**Objective:**

To analyse AKI severity/resolution and laboratory aspects of paediatric patients with DKA managed with 0.9 sodium chloride boluses in larger initial volumes than recommended by current international guidelines.

**Methods:**

Retrospective cohort study conducted at the Emergency Department (ED) of a tertiary paediatric hospital in Brazil. Medical records of children and adolescents with DKA were reviewed from January 2008 to December 2017. Severity of AKI was assessed according to the Kidney Disease: Improving Global Outcomes (KDIGO) criteria. Laboratory data for 72-hours and time to DKA and AKI resolution were analysed. Linear regression models and survival analysis of time to DKA and AKI resolution were performed.

**Results:**

Sixty-eight DKA episodes were included, and 61 had a kidney function assessment. The AKI prevalence was 76%: 48% stage-1, 28% stage-2. No patients developed stage-3 AKI. Patients received an average of 35± (16.0) mL/kg 0.9% sodium chloride bolus in the first 2 h of treatment and 54± (21.7) mL/kg in the first 4-hours. The mean time to AKI resolution was 10.4 (±15.0) hours: stage-1 AKI resolution was 8.1 (±9.0) hours, and stage-2 resolution was 24.9 (±20.1) hours (*p* = 0.001). Forty-three percent of severe DKA episodes were associated with stage-1 AKI and 52% with stage-2 (*p* = 0.002). Serum chloride levels were not associated with time for AKI or DKA resolution.

**Conclusion:**

In this single-centre cohort, an aggressive initial fluid protocol was associated with rapid AKI resolution without severe hyperchloraemia. However, due to the lack of a control group and potential confounding by indication, these findings require validation in prospective, controlled studies.

## Highlights

• Acute Kidney Injury (AKI) is a common complication of Diabetic Ketoacidosis (DKA), and it may lead to long-term kidney compromise.
• Treatment of DKA with a more aggressive initial fluid therapy is potentially safe and might be associated with renal outcome.
• Hyperchloremia associated with a more aggressive initial fluid therapy with NaCl 0.9% in DKA was not found.
• Different fluid therapy protocols than those recommended by international guidelines for treating patients with DKA should be considered to improve AKI.

## Introduction

The mainstays of Diabetic Ketoacidosis (DKA) treatment are volume replacement, insulin therapy, electrolyte monitoring and replacement, and control of precipitating events. The optimal fluid replacement regimen is controversial, due to its potential involvement in complications.^1^

In 2018, the Paediatric Emergency Care Applied Research Network (PECARN) published the results of the Fluid Therapies Under Investigation in DKA (FLUID) trial, undermining the hypothesis that higher volume infusion rates are associated with cerebral oedema in DKA, it leaves some unanswered questions about Acute Kidney Injury (AKI), which will be addressed in the present study.^2^

In the hospital, an alternative treatment protocol has been used since 1996, with favourable outcomes. It aims to maintain efficiency and patient safety during treatment at the Emergency Department (ED) while optimising resource use, considering a setting with restricted intensive care unit bed availability.^3^ The present protocol differs from the currently recommended by the International Society for Paediatric and Adolescent Diabetes (ISPAD) in regard to a more aggressive initial hydration (first 4‒6 h), exclusive use of 0.9% sodium chloride, less frequent use of maintenance fluids, early transition to oral intake, and subcutaneous insulin instead of intravenous.^1^^,^^4^

The prevalence of AKI in DKA is variable in the literature .^5^, ^6^, ^7^, ^8^, ^9^, ^10^ Menna et al.^11^ A recent systematic review found prevalence of 47% (95% CI 40‒55). Recent studies have emphasised the importance of AKI as a complication of DKA, with sustained negative effects for patients .^7^^,^^12^, ^13^, ^14^ Other studies suggest that high chloride-containing fluids could lead to hyperchloraemia and worse outcomes .^15^, ^16^, ^17^ However, few studies have assessed whether IV fluid boluses or rates of rehydration influence the resolution of AKI during episodes of DKA .^10^^,^^18^^,^^19^ Hence, this study aims to analyse AKI severity/resolution and laboratory aspects of paediatric patients with DKA managed with 0.9% sodium chloride boluses in larger initial volumes used at “Instituto da Criança e do Adolescente ‒ HCFMUSP”.

## Research design and methods

Retrospective cohort study conducted at the ED of a tertiary paediatric hospital in Brazil, following the STROBE statement. The study was approved by the Ethics Committee (approval number 88,514,218.6.0000.0068), and informed consent was dismissed since an anonymised database from previous studies was used.

Cases of DKA were identified using the International Classification of Diseases, Tenth Revision (ICD-10) codes for diabetes (E10–14) from January 1st, 2008, to December 31st, 2017. Patients were eligible for inclusion in the study if they were 18-years of age or younger and had confirmed DKA (blood glucose level > 200 mg/dL, pH < 7.3, or bicarbonate level < 15 mEq/L, and elevation of serum or urine ketones) .^1^

In the studied hospital, the DKA treatment protocol consists of initiation of fluid replacement with a 0.9% sodium chloride solution at 20 mL/kg/hour (maximum 1.000 mL/hour). Additional boluses of normal saline (10–20 mL/kg) are administered hourly for 4 to 6 h if capillary refill time remains greater than 3 s. Maintenance fluid is administered only if oral fluids are not tolerated. Potassium replacement is initiated after the first hour of treatment, with either potassium chloride or phosphate according to serum electrolyte levels. Insulin therapy is initiated after the first hour with a subcutaneous rapid-acting insulin analogue .^3^

Medical records of patients with DKA were reviewed. Clinical and biochemical assessments, including hourly Glasgow Coma Scale evaluation, neurological signs, fluid and insulin administration, were recorded until 72-hours after ED admission.

Patients with incomplete medical documentation, other underlying medical conditions, or who received hypotonic fluids were excluded.

For the evaluation of acute kidney injury, the Kidney Disease/Improving Global Outcomes (KDIGO) serum creatinine criteria were used.^20^:

• Stage-1 AKI (KDIGO-1): Serum creatinine is 1.5 to 1.9 times baseline or has an increase over 0.3 mg/dL in < 48 h.
• Stage-2 AKI (KDIGO-2): Serum creatinine is 2 to 2.9 times baseline.
• Stage-3 AKI (KDIGO-3): Serum creatinine is 3-times baseline or has an increase over 4 mg/dL or initiation of renal replacement therapy or decrease of glomerular filtration rate to <35 mL/min per 1.73 m.^2^

The baseline creatinine was considered the most recent creatinine level before the DKA episode. When this was unavailable, an estimated Glomerular Filtration Rate (GFR) of 120 mL/min/1.73 m^2^ was used to calculate an expected baseline creatinine level using the Schwartz estimating equation .^21^ This criterion was selected based on previously established standards in paediatric AKI studies .^22^, ^23^^,^^24^

Corrected sodium (Na) was calculated using the formula: Corrected Na = measured Na + 1.6(.[plasma glucose– 100]/100) mg/dL .^25^

### Statistical analysis

Continuous variables were described as means and standard deviation or as medians (range or IQR), according to the Shapiro-Wilk test. Means or medians were compared using either ANOVA or the Kruskal-Wallis-Dwass-Steel-Critchlow-Fligner pairwise. Categorical variables were described in percentage terms and compared using Fisher’s exact test.

Random-effects linear regression models were used to assess the effects of AKI severity and DKA severity over time on each of the dependent variables (repeated measures). The random effect in the linear regression models was the patient ID to account for repeated episodes. Once differences in means were detected, distinct groups of means were identified through multiple comparisons with Bonferroni correction. Data normality was assessed using the Kolmogorov-Smirnov test.

A group-based trajectory model was applied to identify patterns of similarity in patients’ chloride levels across the nine evaluation time points.

Survival analysis evaluated time to event occurrence (AKI and DKA resolution), considering AKI severity, pattern of chloride evolution, and DKA severity. Initially, survival functions were analysed separately for each predictor variable (univariate analysis). Survival functions for each level of these variables were estimated using the Kaplan-Meier model and compared using the Log Rank test (Mantel-Cox). Univariate and multivariate Cox models were fitted to assess the effects of predictive variables on survival time.

The statistical analyses were conducted in SPSS 20.0 and STATA 17 software, with a significance level of 5%.

## Results

During the study period, a total of 233 children with diabetes visited the ED; 97 were diagnosed with DKA and met the criteria for analysis. Twenty-nine cases were excluded due to incomplete documentation (14), hypotonic fluid infusion (12), or other underlying medical conditions (3).

Sixty-eight DKA episodes were included in the final analysis; 14 (20.5%) corresponded to newly diagnosed type-1 diabetes. Twelve patients (17.6%) had more than one DKA episode, with at least a two-month difference. Twenty-four episodes (35%) were in male patients and 44 (65%) in female patients. The mean age of patients was 12-years (±3.4), ranging from 3 to 17-years of age. No patients presented with cerebral oedema or required neurological intervention.

In 61 episodes, kidney function was assessed (39 patients had previous creatinine results, 22 were estimated and seven episodes had no records of the patient’s height, making the Schwartz equation infeasible). AKI was present in 42 (69%) episodes at ED admission, and four patients developed AKI within the first hours of treatment.

Table 1 highlights findings according to the maximum AKI stage during admission. Fifteen patients (25%) did not develop AKI, 29 (48%) presented with stage-1 and 17 (28%) with stage-2. No patients developed stage-3 AKI. It was observed that stage-2 AKI at ED admission was more associated with moderate and severe DKA.

**Table 1:** Characteristics of 61 children aged 18-years or younger with type 1 diabetes, grouped by maximum acute kidney injury stage during admission.

Characteristics	No AKI, n = 15 (25%)	Stage-1, n = 29 (48%)	Stage-2, n = 17 (28%)	p-value
Total 61 episodes				
Patient demographic characteristics				
Age	12.6 (±3.02)	12.0 (±3.79)	12.1 (±3.19)	0.83
Males, n (%)	5 (33)	10 (34)	7 (41)	
New diagnosis of DM 1	2 (13)	6 (21)	3 (18)	0.91
AKI at admission	0	25 (86)	17 (100)	0.001^c^
				
Fluid boluses (mL/kg)				
2h	35.0 (±14.0)	33.0 (±17.0)	36.0 (±17.0)	0.82
4h	53.0 (±22.2)	52.2 (±24.0)	58.0 (±19.0)	0.64
6h	58.0 (±22.0)	61.0 (±26.0)	63.0 (±16.0)	0.53
12h	60.0 (±24.0)	65.0 (±31.0)	65.2 (±20.0)	0.43
				
Potassium replacement				0.63
Potassium chloride	11 (73)	18 (62)	11 (65)	
Potassium phosphate	4 (27)	9 (31)	6 (35)	
Both solutions	0	2 (7)	0	
				
Findings				
*DKA severity*				0.003
Mild	6 (40)^a^	13 (45)^b^	1 (6)^a^^,^^b^	
Moderate	8 (53)^a^	7 (24)^b^	5 (29)^a^^,^^b^	
Severe	1 (7)^a^	9 (31)^b^	11 (65)^a^^,^^b^	
Mod/severe	9 (60)^a^	16 (55)^b^	16 (94)^a^^,^^b^	
Time to AKI resolution (hours)	-	4 (2‒36)	24 (2‒48)	0.003
Time to DKA resolution (hours)	6 (2–36)	12 (4‒24)^b^	14 (4‒36)^b^	0.01
				
Initial Laboratory values				
pH	7.23 (7.11‒7.31)^a^	7.20 (6.93‒7.31)^b^	7.07 (6.90‒7.27)^a^^,^^b^	<0.001
Serum Bicarbonate (mEq/L)	10.9 (±3.5)^a^	10.1 (±3.9)^b^	6.8 (±3.1)^a^^,^^b^	0.002
Anion gap	34.2 (±6.0)	38.4 (±8.2)	40.5 (±8.3)	0.05
Corrected Sodium (mEq/L)	138 (±5.0)	139 (±4.4)	138 (±4.7)	0.81
Potassium level (mEq/L)	4.8 (3.9–6.2)^a^	5.25 (3.7‒8.9)^b^	5.3 (4.5‒7.6)^a^^,^^b^	0.03
Chloride level (mEq/L)	97.0 (±3.1)	94 (±6.0)	95 (±8.1)	0.49
Phosphorus level (mEq/L)	3.9 (2.2–6.2)^a^	5.2 (2.7‒10.0)	6.2 (3.6–9.1)^a^	0.003
Serum Creatinine level (mg/dL)	0.8 (±0.2)^a^	0.9 (0.3)^b^	1.3 (±0.2)^a^^,^^b^	<0.001
Serum Urea Nitrogen level (mg/dL)	32.0 (±11.0)^a^	35.5 (±10.4)^b^	45.0 (±12.4)^a^^,^^b^	0.02

n (%); mean (±SD); median (min‒max).

a Group KDIGO 0 e 2.

b Groups KDIGO 1 e 2.

c n (%) Fisher test. Mean - ANOVA-Tukey. Median - Kruskal-Wallis -Dwass-Steel-Critchlow-Fligner pairwise comparisions.

Mean time to AKI resolution was 10.4 (±15) hours. Stage-1 AKI resolved in 8.1 (±9) hours while stage-2 AKI lasted 24.9 (±20.1) hours (*p* = 0.001). Patients with stage-2 AKI had longer median time to AKI (24-hours: 2‒48) and DKA (14-hours: 4‒36) resolution. Regarding laboratory tests at admission, serum pH and bicarbonate values were lower in patients with stage-2 AKI, while potassium, phosphorus and urea were higher in this group of patients. There was no difference in baseline chloride in relation to kidney function at admission. The mean volume of saline received by patients was 35 (±16) mL/kg at 2-hours and 54 (±21.7) mL/kg at 4-hours. Patients with stage-2 AKI received more fluids at 6- and 12-hours than patients without AKI and similar to stage-1.

As for DKA severity at admission, 23 (34%) patients had mild DKA (pH > 7.2), 21 (31%) had moderate (pH 7.1‒7.2) and 24(35%) had severe (pH < 7.1). Table 2 summarizes the findings according to DKA severity at presentation. The mean volume of isotonic fluid received by patients at 4-hours was not statistically different between the groups (*p* = 0.08). However, at 6-hours of treatment, more severe DKA was associated with larger fluid boluses: 53.2 (±18.1) mL/kg in mild vs. 73.4 (±26.0) mL/kg in severe episodes (*p* = 0.01).

**Table 2:** Characteristics of 68 children aged 18-years or younger with type 1 diabetes, grouped by diabetic ketoacidosis severity at hospital admission.

Characteristics	Mild DKA (n = 23)	Moderate DKA (n = 21)	Severe DKA (n = 24)	p-value
Total 68 episodes				
Patient demographic characteristics				
Age	11.4 (±3.4)	12.5 (±3.4)	12.1 (3.5)	0.56
New diagnosis of DM 1	8 (35)	2 (10)	4 (17)	0.10
				
Fluid boluses (mL/kg)				
2h	30.4 (±10.6)	36.3 (±12.1)	39.1 (±22.74)	0.12
4h	48.2 (±16.81)	56.6 (±17.0)	62.7 (±29.1)	0.08
6h	53.2 (±18.1)^a^	62.4 (±19.2)	73.4 (±26.0)^a^	0.01
12h	56.1 (±20.5)^a^	62.9 (±19.7)^b^	80.4 (±31.2)^a^^,^^b^	0.01
				
Potassium replacement				0.61
Potassium chloride	14 (61)	14 (67)	18 (75)	
Potassium phosphate	8 (35)	7 (33)	5 (21)	
Both solutions	1 (4)	0	1 (4)	
				
Findings				
AKI on admission	12/20 (60)	11/20 (55)^b^	19/21 (91)^b^	0.03
*Maximum AKI stage*				0.002
No AKI	6/20 (30)^a^	8/20 (40)^b^	1/21 (5)^a^^,^^b^	
Stage-1	13/20 (65)^a^	7/20 (35)^b^	9/21 (43)^a^^,^^b^	
Stage-2	1/20 (5)^a^	5/20 (25)^b^	11/21 (52)^a^^,^^b^	
Time to AKI resolution (hours)	4 (2‒12)^a^	15 (2‒48)	12 (2‒48)^a^	0.04
Time to DKA resolution (hours) pH ≥ 7.3 e bic ≥ 15 (hours)	6 (2‒24)^a^	12 (4‒24)^b^	16 (10‒36)^a^^,^^b^	<0.001
	
Initial Laboratory values				
pH	7.26 (7.21‒7.31)^a^	7.17 (7.10‒7.25)^b^	7.03 (6.90‒7.11)^a^^,^^b^	<0.001
Serum Bicarbonate (mEq/L)	13.8 (±1.6)^a^	9.3 (±2.0)^b^	5.6 (±2.1)^a^^,^^b^	<0.001
Anion gap	32 (±5.9)^a^	37.2 (±6.1)^b^	43.2 (±7.0)^a^^,^^b^	<0.001
Corrected Sodium (mEq/L)	138.1 (±4.6)	137.2 (±3.1)	139.3 (±5.4)	0.253
Osmolality (mOsm/kg)	740 (±125)	715 (±107)	786 (±177)	0.267
Potassium level (mEq/L)	4.7 (3.7‒5.5)^a^	5.1 (4.0‒6.2)^b^	5.6 (4.5‒8.9)^a^^,^^b^	<0.001
Chloride level (mEq/L)	96.4 (±4.8)	95.3 (±5.3)	95.8 (±7.7)	0.775
Phosphorus level (mEq/L)	4.4 (2.7‒7.5)^a^	5.4 (2.2‒8.0)	5.9 (3.5‒10.0)^b^	0.001
Serum Creatinine level (mg/dL)	0.82 (±0.21)^a^	0.93 (±0.23)^b^	1.17 (±0.31)^a^^,^^b^	<0.001
Serum Urea Nitrogen level (mg/dL)	31.0 (±8.7)^a^	36.0 (±9.4)	43.5 (±13.1)^a^	0.002

n (%); mean (±SD); median (min‒max).

a Group KDIGO 0 e 2.

b Groups KDIGO 1 e 2. ^c^ n (%) Fisher test. Mean - ANOVA-Tukey. Median - Krusskal-Wallis -Dwass-Steel-Critchlow-Fligner pairwise comparisions.

Ninety-one percent of severe DKA episodes had stage-2 AKI on admission. The median time to AKI normalization was higher in severe DKA compared to mild DKA. As to electrolyte levels and replacement, even though there was a statistically significant difference in phosphorus levels between the groups, the use of chloride or phosphate solution for potassium replacement was similar in all groups, and there was no difference in serum chloride level.

**Fig. 1:**
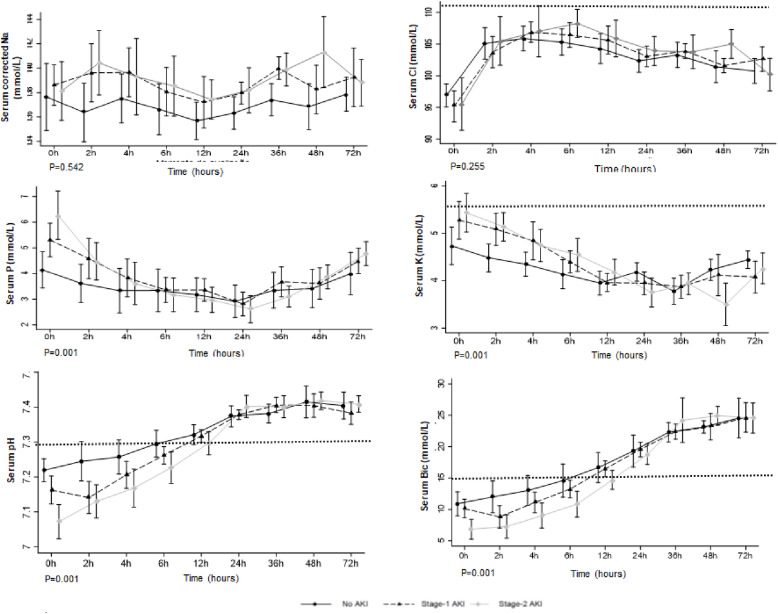
Mean of corrected sodium, chloride, phosphorus, potassium, pH, bicarbonate and anion gap over time, according to AKI severity.

Fig. 1 displays the progression of corrected sodium, chloride, phosphorus, potassium, pH, and bicarbonate means of each group according to the level of kidney dysfunction through time, during DKA treatment. Neither corrected sodium (*p* = 0.742) nor chloride (*p* = 0.547) had a statistically significant difference related to AKI stages. Serum chloride had a considerable increase during the first two hours of treatment, reaching a maximum value between 4- and 12-hours of admission. Nevertheless, pH and bicarbonate improved even while chloride rose.

**Fig. 2:**
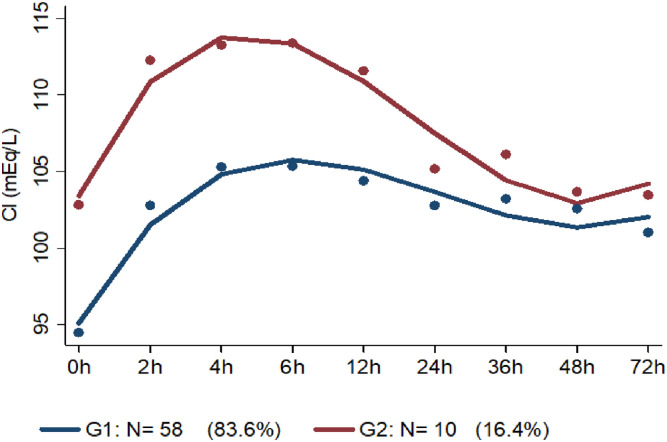
Results of group-based trajectory models on chloride variation.

Fig. 2 shows the results of the chloride patterns using a group-based trajectory model. This analysis resulted in the identification of two distinct groups based on serum chloride variation. No patients had chloride over 112 mEq/L at 24-hours.

**Fig. 3:**
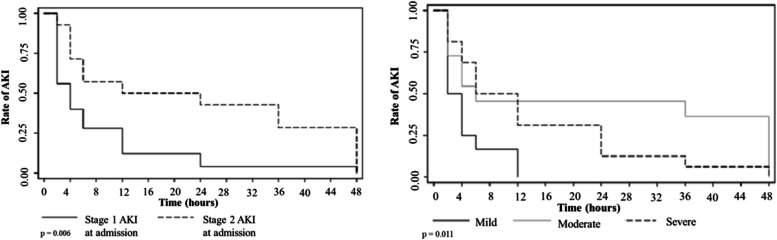
Survival analysis of AKI resolution according to AKI severity (left) and DKA severity (right).

Fig. 3 represents the survival analysis of the resolution of AKI according to AKI level and DKA severity. Both AKI degree and DKA severity influenced the time to normalization of renal function. The Cox models showed fluid volume received in the first 6-hours was not an independent predictor of AKI or DKA resolution and no association was found between chloride variation in the first 6-hours of treatment or chloride variation group and DKA and AKI resolution.

All episodes showed a return to baseline creatinine within 48 h. After 12 h of admission, only 12% of stage-1 patients maintained AKI, while 50% of stage-2 remained with renal dysfunction. After 24-hours of admission, around 96% of stage-1 episodes showed normalization of renal function, while only 5% of stage-2 episodes achieved this goal. Thirty-six hours after admission, 71% of stage-2 episodes showed normalization of renal function.

Considering DKA severity, after 12-hours of admission, all episodes with mild DKA showed normalization of renal function, while 46% with moderate DKA and 31% with severe DKA maintained AKI. Normalization of renal function of all episodes happened within 48-hours. There was no difference in time to AKI and DKA resolution between the two chloride pattern groups.

Seven patients had neurologic symptoms during DKA treatment (2 had headache and 5 somnolence). Of these, 2 had no AKI, 3 had stage-1 and −2 had stage-2. No patients required intervention or presented evolution compatible with cerebral oedema. No follow-up evaluation of cognitive function was performed.

## Discussion

There has been an increasing number of studies published over the past five years focusing on AKI in children with DKA. However, few studies have evaluated whether Intravenous (IV) fluid bolus volumes or rehydration rates influence AKI resolution during DKA episodes. In the present study, an aggressive initial isotonic fluid protocol was used and there was a rapid AKI resolution without severe hyperchloremia.

The authors observed a high prevalence of AKI (76%), which exceeds rates reported in several previous studies .^5^, ^6^, ^7^, ^8^, ^9^, ^10^, ^11^ A key and distinguishing finding, however, was that all patients recovered their baseline kidney function within 48-hours of admission. This rapid reversal is a clinical hallmark of prerenal AKI and suggests an effective response to the isotonic fluid therapy administered at the studied institution. Notably, 15% of episodes presented without AKI at admission, and 63% of AKI cases were classified as KDIGO stage-1 or −2. This distribution may reflect facilitated access to medical care through Brazil’s Unified Health System (SUS), allowing for earlier presentation and prompt intervention.

The treatment approach was beneficial for DKA episodes complicated by KDIGO stage-1 and stage-2 AKI. Although it is plausible that patients with stage-3 AKI might also benefit from aggressive volume resuscitation, this could not be assessed because no patients in this cohort presented with or progressed to KDIGO stage-3. Both AKI severity and DKA severity influenced the time to kidney function normalization. Patients with stage 1 AKI or mild DKA demonstrated a faster return to baseline serum creatinine, while those with stage-2 AKI or moderate-to-severe DKA also showed favourable outcomes, with complete kidney recovery within 48-hours and no need for diuretics or kidney replacement therapy. This rapid and complete resolution suggests that the initial fluid strategy used at the institution may have contributed to these positive outcomes, contrasting with some previous reports .^5^^,^^26^ Nevertheless, this finding should be interpreted cautiously, as sicker patients (i.e., those with more severe DKA and stage-2 AKI) received larger fluid volumes at 6- and 12-hours, reflecting clinical decisions to address greater hypovolaemia. Thus, favourable outcomes were observed in a specifically and aggressively treated cohort, and further studies are needed to validate these findings and compare them with the ISPAD-recommended approach.

An interesting observation was that AKI resolution time was longer in moderate DKA than in severe DKA. Patients with moderate DKA received lower fluid volumes at 6- and 12-hours compared with those with severe DKA (62.4 ± 19.2 mL/kg vs. 73.4 ± 26.0 mL/kg and 62.9 ± 19.7 mL/kg vs. 80.4 ± 31.2 mL/kg, respectively; *p* = 0.01). However, Cox proportional hazards models demonstrated that fluid volume administered within the first 6-hours was not an independent predictor of AKI or DKA resolution. One possible explanation for the longer resolution time in moderate DKA is underestimation of dehydration severity in this group, which may have delayed optimal volume resuscitation and compromised outcomes in the first 48-hours.

Similar to these findings, Hursh et al.^26^ reported a 64% prevalence of AKI in a single Canadian centre, higher than that reported in other studies,^5^^,^^27^ although 19.8% of their cases were classified as AKI stage-3. The authors noted that most patients (75%) had newly diagnosed type-1 diabetes at presentation, which may complicate early diagnosis and contribute to delays in seeking medical care. AKI severity was associated with DKA severity in that cohort. While the authors also observed an association between AKI and DKA severity, as reflected by pH and bicarbonate levels, they found no differences in corrected sodium at admission or over time, unlike the findings reported by Hursh et al.^26^ and Huang et al.^5^ In the present study, potassium and phosphorus were the electrolytes most strongly associated with kidney dysfunction.

Bergman et al.^10^ evaluated AKI in children with DKA who received either < 15 mL/kg or 15–20 mL/kg of IV 0.9% saline as an initial fluid bolus. The prevalence of AKI was 25%, lower than that observed in the cohort. However, approximately 8% of encounters showed persistent AKI at 48 h, whereas all episodes in the present study demonstrated complete AKI resolution within this timeframe.

The association of low serum pH and bicarbonate levels with tachycardia, reported in previous studies .^5^^,^^26^, suggests that severe dehydration and acidosis may contribute to kidney injury. Accordingly, more intensive early fluid therapy may favor AKI resolution. In the present cohort, patients with moderate and severe DKA received higher fluid volumes within the first 6–12 h, which may have contributed to the observed improvement in kidney function.

Although serum chloride levels increased during the initial hours of treatment, this was not associated with delayed DKA resolution or worsening of AKI. Rewers et al.^28^ reported similar findings in a post hoc analysis of the FLUID trial. In this study, an aggressive initial fluid protocol was associated with rapid AKI resolution without severe hyperchloraemia or delayed DKA resolution due to hyperchloraemic acidosis. Ahmed et al.^16^ and Balaji et al.^17^ reported that serum chloride levels > 112 mEq/L at 24-hours were associated with AKI (73.3% sensitivity and 82.4% specificity). Notably, none of our patients had chloride levels exceeding 111 mEq/L at that time point, even when receiving fluid volumes above those recommended in current guidelines.

Patients in the present study received a higher initial fluid bolus (mean 63±23 mL/kg in the first 6-hours). However, early transition to oral intake and discontinuation of IV fluids resulted in a total 24-hour fluid volume of 81.5 ± 40 mL/kg, which may explain the observed chloride trends over time and the absence of severe hyperchloremia.

This study is among the few to evaluate serum phosphorus in children with AKI and DKA and to assess the use of potassium phosphate instead of potassium chloride for electrolyte replacement. Phosphorus and potassium showed similar temporal patterns: elevated at admission, declining with treatment, and stabilising within 72-hours. While all patients received potassium supplementation, only 22 received potassium phosphate. Van der Vaart et al.^29^ also described a similar “U-shaped” pattern of phosphorus levels in children with DKA, without clinically significant consequences of hypophosphataemia.

Because potassium replacement is a cornerstone of DKA management and several studies, including ours .^2^^,^^15^, ^16^, ^17^, suggest lower chloride elevation with potassium phosphate, and its use as an alternative to potassium chloride should be considered.

This study has some limitations: First, this single-centre, retrospective study included a limited number of DKA episodes. As with all retrospective analyses, results depend on the accuracy and completeness of medical records.

Second: Baseline creatinine values were unavailable for all patients; therefore, an estimated baseline eGFR of 120 mL/min/1.73 m^2^ was used in 22 episodes. None of the patients with newly diagnosed diabetes had a previous creatinine measurement, which made this estimation necessary since excluding these patients could bring bias to the analysis by considering only patients with a previous DM1 diagnosis and possibly some degree of renal damage. Additionally, the absence of specific kidney injury biomarkers precluded the evaluation of subclinical or long-term renal damage.

Third: The sample size of 68 DKA episodes limits statistical power to assess rare outcomes such as cerebral edema (incidence ∼0.5%–1%), and thus, the safety of this protocol with respect to cerebral edema cannot be definitively established.

Nevertheless, to our knowledge, this is the first study evaluating AKI in DKA using an alternative fluid replacement strategy that also addresses phosphate supplementation.

## Conclusion

In this single-centre cohort, an aggressive initial fluid protocol was associated with rapid AKI resolution without severe hyperchloraemia. However, given the absence of a control group and the potential for confounding by indication, these findings should be confirmed in prospective, controlled studies.

## Authors’ contributions

Bou Ghosn DSN, Farhat SCL, Della Mana T, Schvartsman C: Study conception and design.

Bou Ghosn DSN, Farhat SCL, Ferraz IS: Acquisition, analysis, and interpretation of data.

Bou Ghosn DSN, Farhat SCL, Della Mana T, Schvartsman C: Manuscript drafting and revision.

## Declaration of competing interest

The authors declare no conflicts of interest.

## Data Availability

All data supporting the findings of this study can be obtained from the corresponding author.
